# Management of Open Supracondylar Humeral Fracture in Children: A Case Report and Literature Review

**DOI:** 10.7759/cureus.48119

**Published:** 2023-11-01

**Authors:** Abdallah Boukhentiche, Nazim Benmayouf, Chaouki Derdous, Nouar Boudjouraf, Nacer Khernane

**Affiliations:** 1 Orthopedic Surgery, Benflis Touhami University Hospital, Batna, DZA; 2 Orthopedic Surgery, Batna Specialist Emergency Hospital, Batna, DZA

**Keywords:** kirschner wires, elbow, pediatric fractures, open fractures, supracondylar humeral fracture

## Abstract

Open supracondylar humeral fractures (SHFs) are rare, and there have been few papers specifically addressing their management. In this report, we describe the management and 27-month outcomes of an open SHF. A healthy eight-year-old boy presented with open SHF of the right elbow and underwent percutaneous lateral fixation using two Kirschner wires (K-wire) after irrigation and debridement (I&D) through an anterior approach. Excellent outcomes were obtained without any complications over a 27-month follow-up period. The management of open SHFs in children is yet to be standardized; nevertheless, we believe that I&D is a crucial step, that well-done lateral entry K-wires could provide the required stability, that the anterior approach is logical, safe, and effective, and that the high healing potential of children is our ally in such injuries.

## Introduction

Supracondylar humeral fractures (SHFs) are one of the most common fractures in children [1,2] and have been reported to represent more than 17% of all pediatric fractures [1], with no significant difference in fracture rate by sex [3]. Although SHFs are common in children, open injuries are rare, accounting for 1%, and are more likely to occur in boys and older children, with a significant rate (11.4%) of associated neurovascular injury [3].

Even though open fractures in children have better healing potential than those in adults, the risk of infection, compartment syndrome, and nonunion should not be underestimated [4]. Therefore, understanding the management of open SHFs in children could reduce perioperative complications and optimize long-term results for patients [5]. There have been few reports specifically addressing open SHFs in children [5,6]. In this report, we describe the management and 27-month outcomes of an open SHF in a healthy eight-year-old boy.

## Case presentation

An eight-year-old boy with no significant past medical history was transferred from a local hospital to our department after a fall from a horse about seven hours before admission. He presented with a painful and swollen right arm.

**Figure 1:**
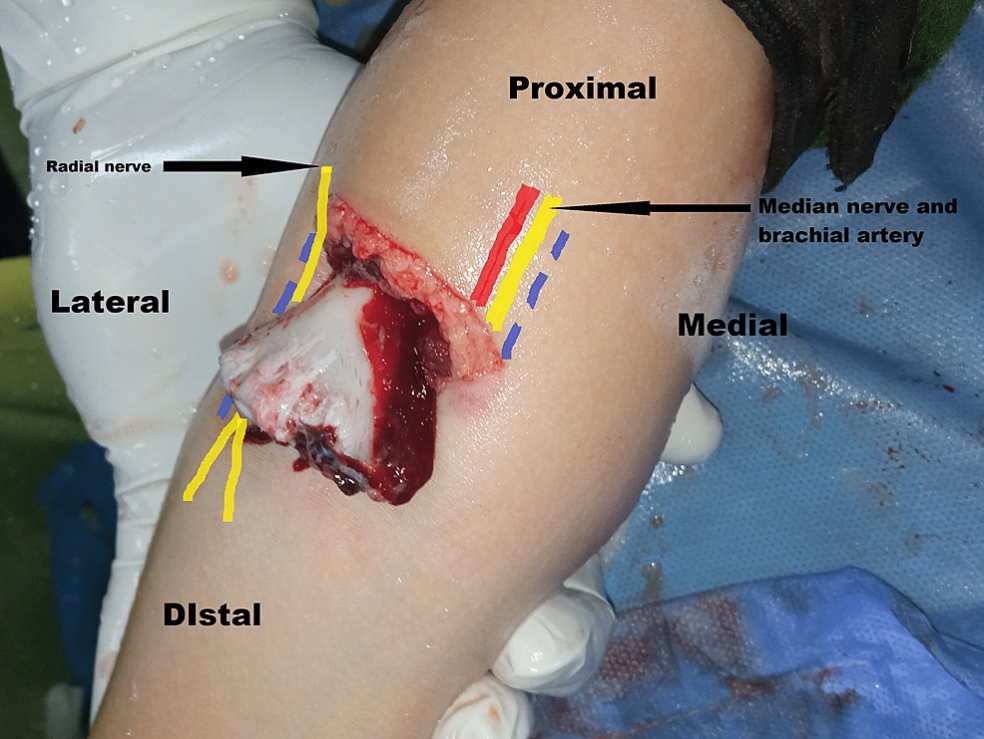
Open supracondylar humeral fracture of the right elbow The distal humerus metaphysis completely emerged through a 4-cm-wide transverse wound on the flexural aspect of the elbow. Landmarks of the anterior approach (blue), median and radial nerve (yellow), and brachial artery (red).

The physical examination revealed a 4-cm-wide transverse wound on the flexural aspect of the elbow, through which the distal humerus metaphysis completely emerged (Figure 1). We assessed the wound without a final staging of the soft tissue injuries until the surgical debridement. The neurovascular assessment was hindered by the anxiety and pain of the patient. However, the hand was warm, the capillary refill time (CRT) was less than three seconds, and the boy was able to move his fingers slightly. A sterile dressing was applied over the elbow, the arm was stabilized by a posterior splint, intravenous antibiotics were administered, and tetanus immunization status was verified.

**Figure 2:**
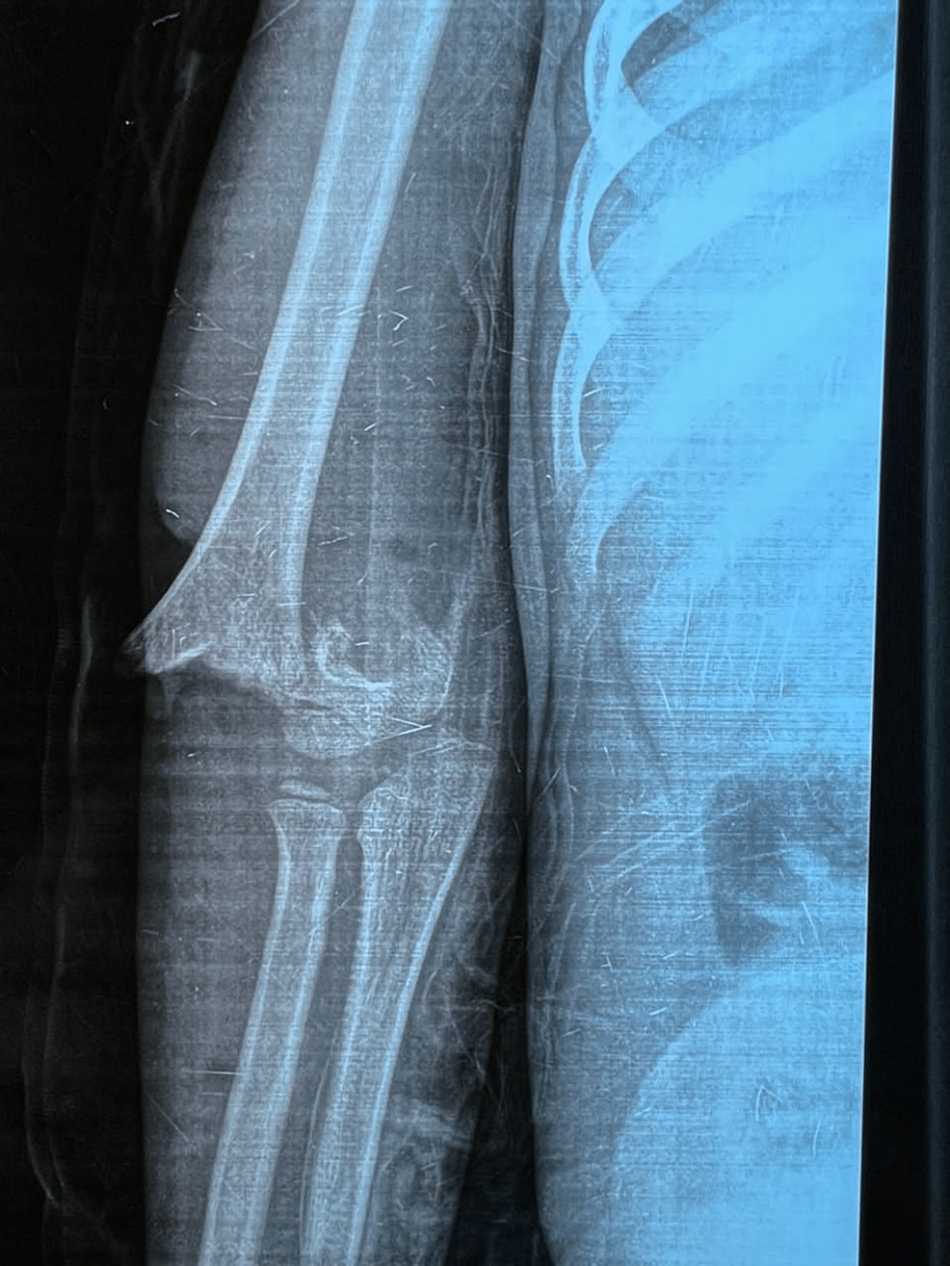
Preoperative radiography of the right elbow A displaced supracondylar humeral fracture that we graded as Gartland extension-type 3.

The plain radiography showed a displaced SHF (Figure 2) that we graded as Gartland extension-type 3 [7]. After preoperative preparation, the patient underwent surgery within one hour.

In the operating room, under general anesthesia, in a supine position, the extremity was placed on a radiolucent arm board. We started by debriding the proximal edge of the wound, then we extended this horizontal traumatic wound by two longitudinal incisions, the first ascending medially and the second descending laterally. Next, by holding up the proximal fragment with a Lambotte bone hook, we were able to continue the debridement of the distal edge of the wound. The surgical site was thoroughly irrigated with an isotonic saline solution. The surgical exploration revealed an intact brachial artery without rupture on the median or radial nerves. The soft tissue injury was staged as Gustilo-Anderson type 2 [8].

The distal fragment was exposed through the disrupted brachialis and torn anterior periosteum, while the posterior periosteum remained partially undamaged. The fracture was reduced by gentle traction on the elbow flexed 20° to 30°. To avoid any damage or entrapment of the soft tissues, they were kept away from the fracture site on either side by two Farabeuf retractors, and the distal edge of the anterior torn periosteum was held out of the fracture site with absorbable sutures. While the elbow was almost straight, we corrected the varus and the valgus angular alignment, and then we completed the reduction by flexing the elbow slowly. The reduction was checked by fluoroscopy images in the anteroposterior, lateral, and oblique planes.

**Figure 3:**
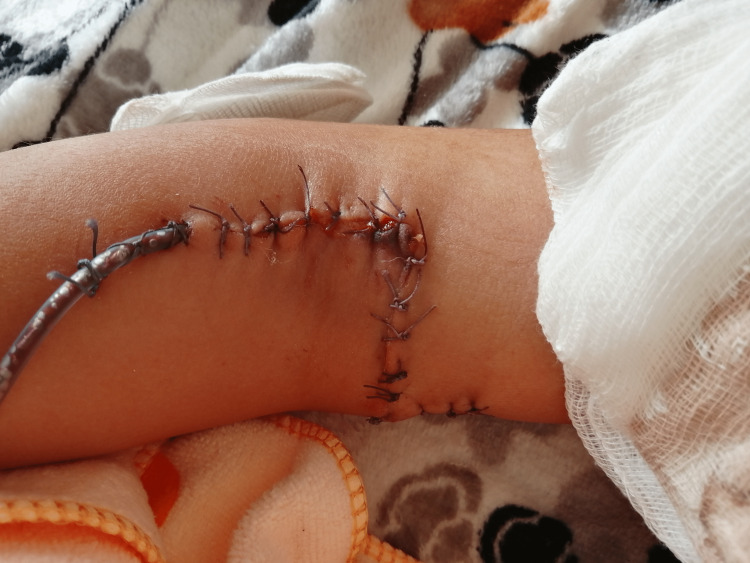
The first postoperative dressing change

Once the reduction was confirmed, the fixation was performed in elbow hyperflexion with two parallel smooth Kirschner wires (K-wires) based laterally under fluoroscopy control. The transverse wound and the extended parts were closed over a drain using absorbable sutures (Figure 3). A posterior splint was applied at 45° of elbow flexion. The child and his parents were instructed to avoid external shoulder rotation.

**Figure 4:**
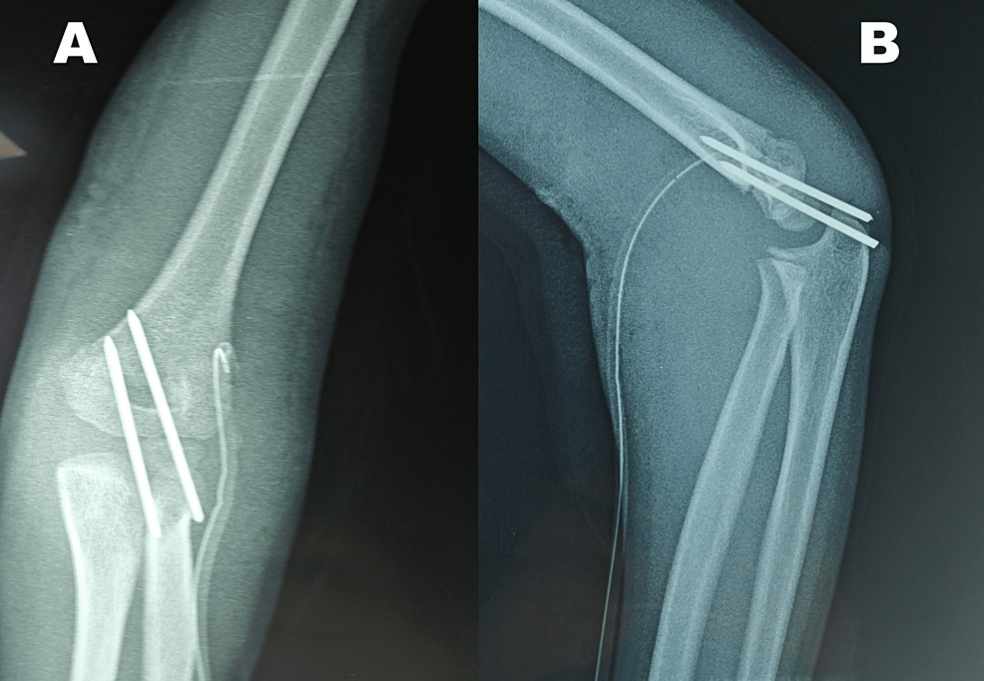
Postoperative radiographs The anteroposterior view (A) shows a bicortical fixation with two parallel K-wires that are sufficiently separated, while the lateral view (B) confirms that the two proximal columns are fixed.

On postoperative radiographs, the anteroposterior view (Figure 4A) showed a bicortical fixation with two parallel K-wires that were sufficiently separated, while the lateral view (Figure 4B) confirmed that the two proximal columns had been fixed [9]. The K-wires were left percutaneous and removed in six weeks.

**Figure 5:**
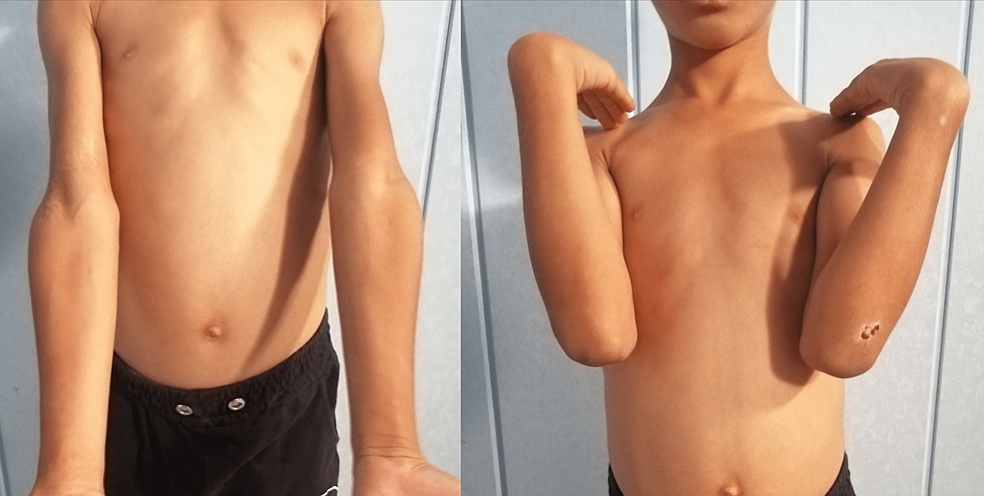
Functional outcomes 27 months after surgery Range of motion of the injured elbow compared to the left elbow

**Figure 6:**
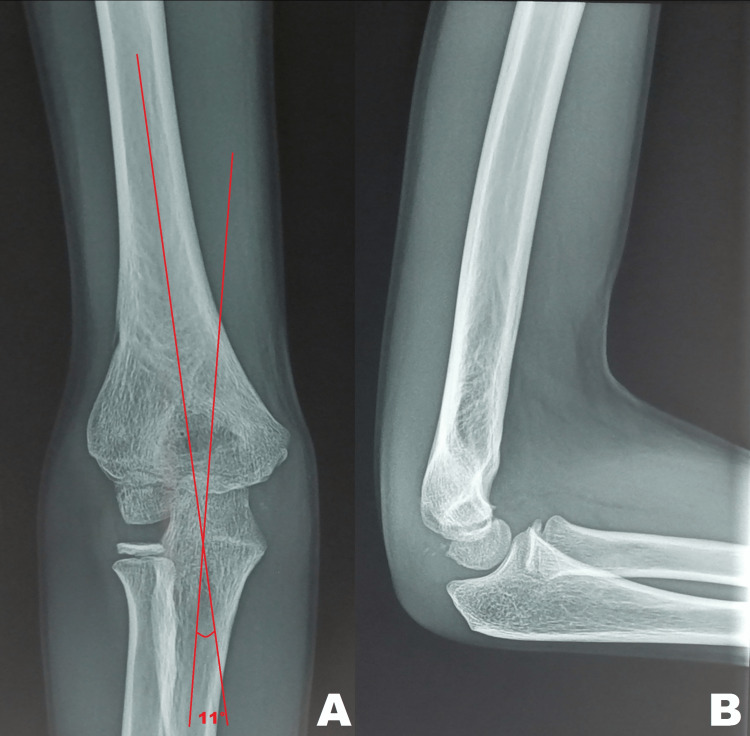
Radiographic outcomes 27 months after surgery Anteroposterior (A) and lateral (B) views 27 months after surgery show a carrying angle of 11° without malunion, osteonecrosis, or myositis ossificans.

The patient was followed up for 27 months without any complications. His last follow-up examination showed a full elbow extension motion and a 138° flexion motion without any deformities (Figure 5). Radiographs revealed a carrying angle of 11° without malunion, myositis ossificans, or osteonecrosis (Figure 6).

## Discussion

The management of SHFs has been well-reported in the literature. Despite some controversy over aspects such as the best K-wire configuration, the consensus is clear about several issues regarding the proper treatment of these fractures [10]. This consensus has been summarized according to Gartland’s classification [7], which is the most widely used. It is a reliable classification with high intra- and inter-observer concordance [11] based on the amount of displacement of the distal fragment.

In contrast, open SHFs in children are often briefly discussed either in the context of open fractures in children or in the context of closed SHFs, but there are few studies that focus specifically on open SHFs [5,6]. As a result, the management protocol of the open SHFs is yet to be standardized. Furthermore, we found three case reports in which management differed from one author to another; while some used a posterior approach and crossed wires [12], others went for external fixation [13,14].

Although these divergent attitudes, basic open fracture principles such as intravenous antibiotics as soon as possible, tetanus prophylaxis as needed, and irrigation and debridement (I&D) remain unchanged. Compared with open fractures in adults, the way in which these principles are applied may sometimes differ because of the high healing potential of children [4,5,12-15]. Stewart et al. reported that the infection rate associated with open SHFs is low as a result of the abundant blood supply around the elbow [4].

Open SHFs are often associated with neurovascular injuries [3]. Additionally, our patient’s neurovascular assessment was quite difficult, so we preferred to extend the anterior transverse wound to check the nerves and vessels and to avoid their iatrogenic damage or entrapment while reducing the fracture [16]. This anterior approach allowed us direct visualization of the brachial artery and median nerve, as well as the fracture fragments. The exposure was through the torn periosteum and disrupted brachialis, which therefore did not further destabilize the fracture. Furthermore, the posterior approach is associated with a high rate of loss of motion and, more importantly, the risk of osteonecrosis of the trochlea [17].

K-wire configuration is one of the controversies in the management of the SHFs [17]. Carrazzone et al. found that crossed wires are more effective at maintaining fracture reduction [18]. Nevertheless, Sankar et al., in a series of 322 fractures, reported that 2.9% (eight patients) had a postoperative loss of fixation. All eight were Gartland type III fractures treated with just two K-wires (seven lateral entries and one crossed wire). In all cases, the loss of fixation was due to technical errors, not to the K-wire configuration itself [19]. Therefore, we opted for lateral-entry K-wires because we believe that, when properly placed, these wires are usually strong enough to maintain reduction even in the most unstable SHFs, as reported in a clinical experience of 124 consecutive SHFs [9].

Twenty-seven months after surgery, the last follow-up showed excellent outcomes according to Flynn's criteria (Table 1) [20], making our management strategy for this patient successful and consistent with what was reported by Stewart et al. that treatment consisting of I&D, fracture reduction, and a lateral entry K-wire was adequate for the vast majority of open SHFs [4]. It is important to note that this paper by Stewart et al. deals with the management of open fractures in children in general and reports the authors' experience with open SHFs in a small paragraph without statistics.

**Table 1:** Criteria for outcomes of supracondylar humeral fracture Compared to the right elbow (145°), the left injured elbow (138°) showed a loss of 7° in flexion and less than 5° of carrying angle loss. The lower of the two values was used to evaluate the outcome according to the criteria of Flynn et al. [20].

Outcome	Cosmetic factor: carrying-angle loss	Functional factor: range of motion loss
Excellent	0–5°	0–5°
Good	5–10°	5–10°
Fair	10–15°	10–15°
Poor	>15°	>15°

## Conclusions

While SHFs are one of the most common fractures in children, open SHFs are rare and poorly reported in the literature. Therefore, their management protocol has yet to be standardized. Controversies such as K-wire configuration and surgical approach remain for both closed and open SHFs. However, adherence to the basic principles of open fracture management is emphasized by all authors, and above all, vigilance must be paid to any potential associated neurovascular injuries. Based on our experience in this case, we recommend the use of two percutaneous lateral K-wires after adequate I&D through an anterior approach for better control of nerves and vessels.
